# Polycomb Group Genes: Keeping Stem Cell Activity in Balance

**DOI:** 10.1371/journal.pbio.0060113

**Published:** 2008-04-29

**Authors:** Martin Sauvageau, Guy Sauvageau

## Abstract

Overexpression of Polycomb group genes is often associated with cancer development, whereas complete deletion results in loss of stem cell activity. New studies show that partial loss of function of Polycomb group genes enhances the activity of blood stem/progenitor cells.

The capacity to undergo self-renewal—to generate daughter cells having the same potency and regenerative properties as the parent—is what defines stem cells. Understanding the molecular mechanisms governing this process remains the holy grail of stem cell biology and holds great promise for the development of stem cell–based therapies aimed at treating debilitating and life-threatening diseases such as cancer. Interestingly, there is support for the idea that several cancers (e.g., blood, brain, breast, melanoma) are made of different cell types, but are driven and sustained mainly by a rare population of “cancer stem cells” that, like normal stem cells, can self-renew and also give rise to non–stem cell progeny. This concept predicts similarities in the genes that regulate self-renewal of normal and cancer stem cells and further emphasizes the importance of identifying the key components regulating these events. Promising candidate genes include the Polycomb group (PcG) family of genes, which play a role in both stem cell self-renewal and in cancer. Although these genes were discovered more than 20 years ago, their function is only slowly being uncovered.

The Polycomb group genes were initially identified as regulators of homeotic genes, master developmental regulators that participate in defining the blueprint for Drosophila's body plan. The identification of similar PcG genes and numerous paralogs in vertebrates raised the intriguing possibility that they may perform similar functions in these organisms (see Table 1 for a full list). In vertebrates, PcG proteins assemble into two discrete chromatin-associated complexes, which have been recently characterized [1–3]. The first complex, referred to as Polycomb Repressive Complex 1 (PRC1), includes at least one paralog of the Pcgf, Ring1, Phc, and Cbx components, whereas the second complex, named PRC2, includes Eed, Ezh, and Suz12, among other proteins. Interestingly, proteins within PRC2 are interdependent, since reduction in any one of them limits the formation of the complex itself [4–6]. PcG protein complexes are mostly associated with heterochromatin, where they maintain gene expression in the off state through histone modifications. The PRC2 proteins Eed, Ezh, and Suz12 form the minimal subunit with enzymatic activity toward histone H3 (methyltransferase activity on lysine 27 of H3 results in H3K27me3) [7,8]. The PRC1 proteins Ring1A/B and Bmi1 show enzymatic activity toward histone H2A (monoubiquityl-ligase on lysine 119 of H2A results in uH2AK119) [9]. These two histone modifications may be coordinated as proposed by the current two-step process model for PcG-mediated repression. In this model, the H3K27me3 covalent mark catalyzed by PRC2 initiates repression and serves as a docking site for the recruitment of the PRC1 complex, resulting in uH2AK119. This covalent modification likely prevents full access to other chromatin remodeling factors or the transcription machinery and facilitates chromatin compaction (see Figure 1) [10–13].

**Table 1:**
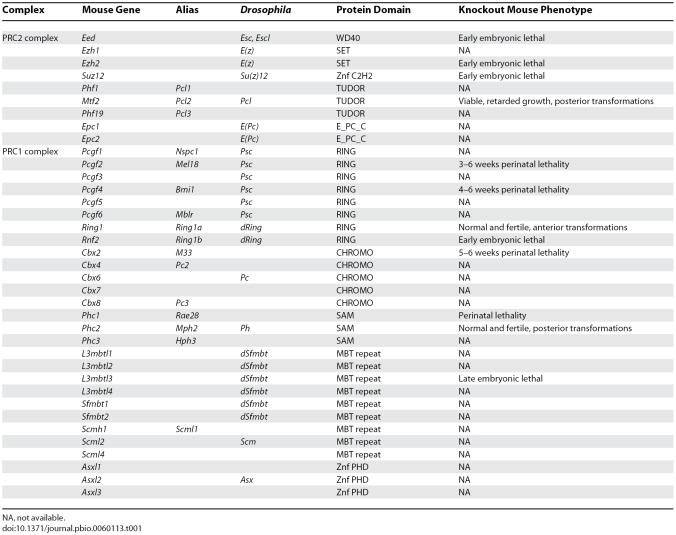
Mouse PcG Knockout Phenotype

**Figure 1:**
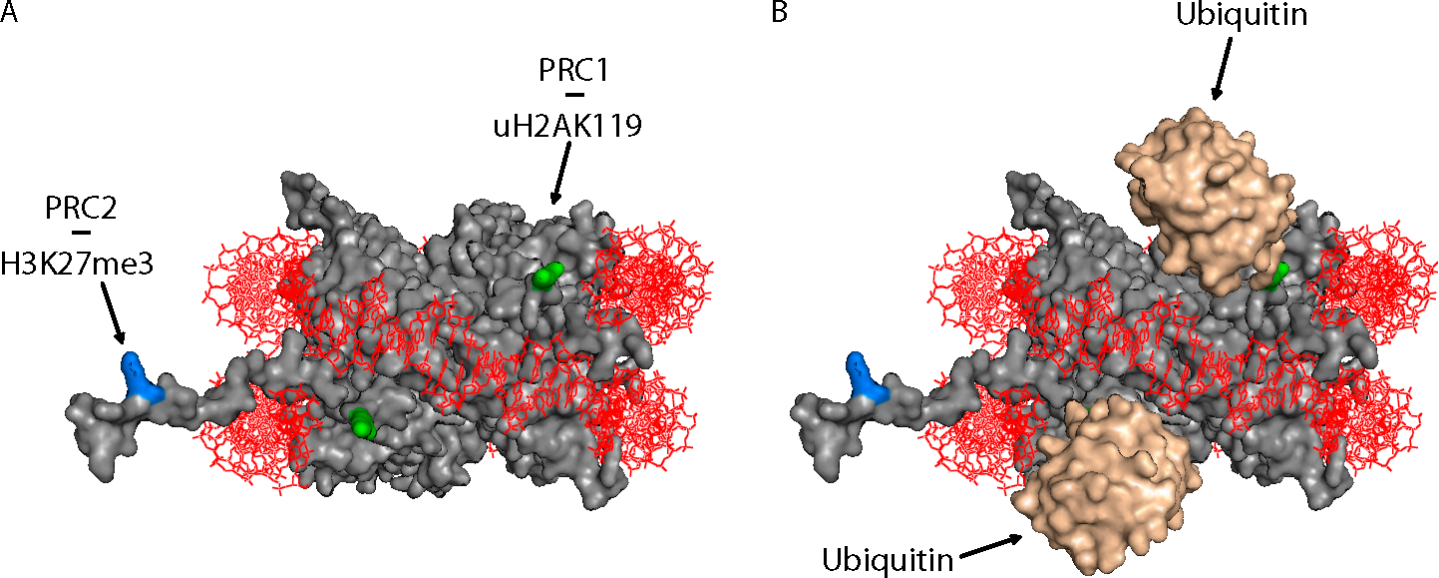
Nucleosome Crystal Structure and Potential Effect of Mono-Ubiquinated H2A on Chromatin Accessibility (A) Representation of the nucleosome crystal structure at 2.8 Å resolution (Protein Data Bank #1AOI) [31]. The histone octamer (in grey) is complexed with 146 base pairs of DNA (red). The histone H3 lysine 27 (blue) located on the N-terminal tail is tri-methylated (H3K27me3) by the PRC2 complex. The histone H2A lysine 119 (green), which is mono-ubiquitylated (uH2AK119) by the PRC1 complex, is located near the entry and exit point of DNA on the histone octamer. (B) In accordance with recent studies, the nucleosome structure shows that, because of their location at the entry and exit point of DNA, ubiquitin molecules (beige) bound to H2AK119 could maintain genes in a repressed state by limiting the access of the RNA polII to chromatin [11]. Interestingly, ubiquitinated H2AK119 is also located at the linker-histone H1 binding region. Studies have shown that uH2AK119 enhances histone H1 interaction with the nucleosome [12,13], suggesting that this epigenetic modification is important for maintaining the compacted chromatin structure.

In mice, loss of function of all core PRC2 components studied to date is embryonic lethal due to severe defects at the implantation and early post-implantation stages (see Table 1). Recently, it was found that embryonic stem (ES) cells mutant for PRC2 genes lose the ability to maintain themselves in an undifferentiated state [14,15]. With the exception of mice mutant for *Ring1b,* which is essential for the survival of early embryos, homozygous null mutant mice for other PRC1 genes (i.e., *Bmi1*, *Mel18*, *Cbx2*, or *Phc1*) survive to birth, but all display homeotic transformations and die perinatally (see Table 1). Functional redundancy and compensation by paralogous genes may explain the milder phenotypes found with most PRC1 versus PRC2 homozygous null mutant mice.

Both PRC1 and PRC2 genes are implicated in regulation of stem cell self-renewal and in cancer development (reviewed in Sparmann et al. [10] and Rajasekhar et al. [16]). *Bmi1* was first discovered as an oncogene overexpressed in lymphomas and cooperating with *c-Myc* [17]. It was found to regulate proliferation and senescence mainly through repression of the *Ink4a* locus [18]. In addition, *Bmi1* is overexpressed in human leukemias and different types of solid cancers [10,16]. This gene also represents an essential regulator of self-renewal for both normal and leukemic hematopoietic stem cells (HSCs), since both of these cell types eventually disappear in its absence [19]. Similar phenotypes were observed with the loss of function of *Phc1,* another PRC1 gene [20].

The PRC2 genes *EZH2* and *SUZ12* are also overexpressed in a broad spectrum of human cancers [10,16]. Notably, *EZH2* is known as a marker for “aggressiveness” in prostate and breast cancer [21,22]. Moreover, recent studies indicate that overexpression of the Ezh2 protein in mouse HSCs preserves self-renewal activity in serial passages, a condition never observed in unmanipulated HSCs and sometimes referred to as “HSC senescence” [23]. This type of activity may be exploited by tumor cells that overexpress these genes. Although activity of *Ezh2* and *Ezh1* homozygous null HSCs remains undescribed, the data with *Ezh2* overexpression are reminiscent of those recently observed with *Bmi1*, potentially indicating that similar molecular bases (e.g., H3K27 tri-methylation; H2A mono-ubiquitination) underlie PRC1 and PRC2 function in HSCs. However, contrasting with *Ezh2* overexpression, partial loss of function and hypomorphic alleles of its PRC2 partner, *Eed,* restricts the proliferation of lymphoid and myeloid progenitors and antagonizes PRC1 function [24]. Two independent studies have also demonstrated that *Eed* possesses tumor-suppressive activity in the hematopoietic system [25,26]. Therefore, it seems that adequate PcG protein levels and activity are important and greatly affect the ability of cells to excessively self-renew (the result of high PcG levels) or to become transformed (the result of low levels).

In this issue of *PLoS Biology*, a study by Ian J. Majewski et al. [27] further strengthens the notion that PRC2 restricts cellular proliferation. In their study, the authors provide evidence that Suz12 is sensitive to gene dosage in the hematopoietic compartment and that reduction in Suz12 levels enhances the activity of certain hematopoietic cells. By using ENU (N-ethyl-N-nitrosourea) mutagenesis and positional cloning experiments, Majewski et al. [27] identified an inactivating point mutation in *Suz12*, called *Plt8*, which is embryonic lethal in the homozygous state. More importantly, the study showed that heterozygote *Suz12^Plt8/+^* mice are viable and display increased numbers of platelets, megakaryocytes, lymphoid cells, and certain progenitors. Interestingly, the *Plt8* mutation partly rescues the hematopoietic phenotype observed in mice lacking the thrombopoietin receptor c-Mpl. Moreover, the authors show that *Suz12^Plt8/+^* bone marrow cells are more competitive than wild-type counterparts, suggesting a negative regulatory role for Suz12 in HSC activity. The phenotype described in *Suz12^Plt8/+^* mice was reproduced by partial knockdown of *Suz12* using RNA interference, confirming that the mutant phenotype is a result of decreased *Suz12* expression. The authors also showed that *Ezh2* levels are reduced in *Suz12^Plt8/+^* cells and that heterozygotic mutation of *Ezh2* rescues defects seen in *c-Mpl^−/−^* mice similarly to *Suz12^Plt8/+^* mutants. Although further experiments are needed, this suggests that *Ezh2* is also haploinsufficient and that low levels enhance hematopoietic activity.

The study by Majewski et al. [27] is clearly reminiscent of the results seen in partial loss of function of Eed. It indicates that complete loss of PRC2 components is detrimental to cells and produces unviable embryos, but that partial reduction in their levels has the opposite effect and enhances HSC and progenitor cell activity. In the case of *Eed^null/+^* and homozygous hypomorph mutants, this reduction eventually leads to leukemia development [24–26]. Although the authors did not observe any leukemia in *Suz12^Plt8/+^* mice, oncogenic insults and additional mutagenic events may be required for full transformation of *Suz12^Plt8/+^* cells. This hypothesis could also be true for Ezh2 and should be tested. Interestingly, the human chromosomal locations of *EED*, *EZH2*, and *SUZ12* are all found in regions of recurrent chromosomal deletions and aberrations. *EED* is particularly interesting because it is located in close proximity to *ATM* and *MLL*, two genes frequently involved in hematopoietic malignancies. Irradiation or carcinogen treatment of *Suz12^Plt8/+^* or *Ezh2^+/−^* cells may thus reveal a similar tumor-suppressive function as observed with *Eed* mutant mice.

Together with the current knowledge on Polycomb group genes and their role in self-renewal and cancer, the study by Majewski et al. [27] provides further evidence for a delicate balance and tight regulation of the PRC2 complex levels for proper function of stem and progenitor cells. This leads to a gene dosage model where up-regulation or modest down-regulation of the PRC2 complex tips the balance toward enhanced HSC activity and increased chances of developing tumors, whereas complete knockout results in stem cell loss (see Figure 2).

**Figure 2:**
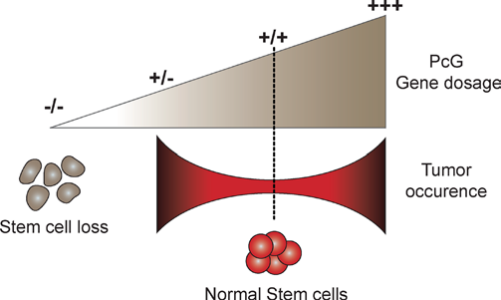
Model for Gene Dosage Effect of PcG Genes on Stem Cells and Cancer Adequate PcG gene levels, such as in wild-type cells (+/+), seem to be crucial for normal stem cell functions. Either overexpression (+++) or partial loss of function (+/−) of PcG genes leads to an increase in tumor development. In turn, complete ablation (−/−) is detrimental and leads to impairment or loss of stem cells.

This model raises many questions regarding the function of Polycomb group genes in stem cell self-renewal and cancer. First, is there a similar dosage effect for PRC1 genes? Human *PHC1* is located on Chromosome 12p13, a region frequently associated with loss of heterozygosity in acute lymphoblastic leukemia [28]. Studies on compound *Bmi1* and *Mel18* mutant mice seem to suggest that these genes are sensitive to dosage variations [29]. Careful analysis of stem cell activity and sensitivity to transformation in heterozygous mice would be of great interest. The mechanisms through which PcG haploinsufficiency versus overexpression leads to cancer are also yet to be defined. Do the results observed occur through similar or distinct pathways? This question is especially relevant now that we know that PcG proteins interact with multiple other proteins and potentially have non-histone substrates, suggesting as yet unknown functions for both PRC1 and PRC2 complexes.

**Glossary**

**Embryonic lethal:** Leading to death of embryos during embryonic development.

**Haploinsufficiency:** When loss of one functional copy in a diploid organism results in a phenotype.

**Homeotic transformation:** Major shift in the developmental fate of an organ or body part, especially to a homologous organ or part normally found elsewhere in the organism.

**Hypomorphic gene:** A mutant gene having a similar but weaker effect than the corresponding wild-type gene.

Taking into account that most cancers are derived from a single cell (clonal), it can be difficult to compare PcG gene expression levels in the rare normal cells in which transformation occurs to that in the cancer stem cells. Tools and knowledge are becoming available to resolve this important issue. Likewise, it is still not clear if PRC2 and/or PRC1 activity is enhanced as a result of PcG gene deregulation in these normal or tumor stem cells. Although a pattern of PcG-mediated histone modifications was recently ascribed to certain stem cells [30], its implication in self-renewal remains difficult to assess. Such an endeavor would require the generation of histone mutants, a technical challenge in vertebrates considering the multiple variants and genes coding for all four nucleosomal subunits. In addition, evidence that PcG proteins also display non-chromatin-related activity raises a fundamental issue about the targets (i.e., nucleosomes versus others) that control self-renewal in cancer and normal stem cells.

Finally, since very little is known about the transcriptional and post-transcriptional regulation of PcG genes, it becomes important to elucidate the pathways that determine the cellular levels of these proteins in order to prevent stem cell loss and cancer development.

## Abbreviations

ES: embryonic stem
HSC: hematopoietic stem cell
PcG: Polycomb group
PRC: Polycomb Repressive Complex
